# Evaluation of multiplex nanopore sequencing for *Salmonella* serotype prediction and antimicrobial resistance gene and virulence gene detection

**DOI:** 10.3389/fmicb.2022.1073057

**Published:** 2023-02-01

**Authors:** Xingwen Wu, Hao Luo, Chongtao Ge, Feng Xu, Xiangyu Deng, Martin Wiedmann, Robert C. Baker, Abigail E. Stevenson, Guangtao Zhang, Silin Tang

**Affiliations:** ^1^Mars Global Food Safety Center, Beijing, China; ^2^Center for Food Safety, University of Georgia, Griffin, GA, United States; ^3^Department of Food Science, Cornell University, Ithaca, NY, United States

**Keywords:** whole genome sequencing, Oxford Nanopore Technologies, *Salmonella*, serotype prediction, foodborne pathogens, food safety, antimicrobial resistance genes, virulence genes

## Abstract

In a previous study, Multiplex-nanopore-sequencing based whole genome sequencing (WGS) allowed for accurate *in silico* serotype prediction of *Salmonella* within one day for five multiplexed isolates, using both SISTR and SeqSero2. Since only ten serotypes were tested in our previous study, the conclusions above were yet to be evaluated in a larger scale test. In the current study we evaluated this workflow with 69 *Salmonella* serotypes and also explored the feasibility of using multiplex-nanopore-sequencing based WGS for antimicrobial resistance gene (AMR) and virulence gene detection. We found that accurate *in silico* serotype prediction with nanopore-WGS data was achieved within about five hours of sequencing at a minimum of 30× *Salmonella* genome coverage, with SeqSero2 as the serotype prediction tool. For each tested isolate, small variations were observed between the AMR/virulence gene profiles from the Illumina and Nanopore sequencing platforms. Taking results generated using Illumina data as the benchmark, the average precision value per isolate was 0.99 for both AMR and virulence gene detection. We found that the resistance gene identifier – RGI identified AMR genes with nanopore data at a much lower accuracy compared to Abricate, possibly due to RGI’s less stringent minimum similarity and coverage by default for database matching. This study is an evaluation of multiplex-nanopore-sequencing based WGS as a cost-efficient and rapid *Salmonella* classification method, and a starting point for future validation and verification of using it as a AMR/virulence gene profiling tool for the food industry. This study paves the way for the application of nanopore sequencing in surveillance, tracking, and risk assessment of *Salmonella* across the food supply chain.

## 1. Introduction

Using the historical data from 1998 to 2019, the recent published report from U.S. CDC has revealed that non-typhoidal *Salmonella* spp. caused most foodborne disease outbreaks and illnesses in the U.S. among four major pathogens including *Listeria monocytogenes*, *E. coli O157*, and *Campylobacter* (Batz et al., 2021; CDC, 2021). Similarly in the report from EFSA, *Salmonella* is the most detected agent and the second-most important cause of foodborne disease cases in the E.U. (EFSA and ECDC, 2021). In addition to the public health risk associated with *Salmonella*, it also imposes significant economic burden on governments and the food industry. To mitigate the risk of *Salmonella* contamination in the supply chain and food production, it is important to have an efficient surveillance and source attribution system. Although more than 2,600 serotypes of *Salmonella* have been identified (Grimont and Weill, 2007), only a small proportion of these serotypes is responsible for the majority of human salmonellosis (CDC, 2013; Jackson et al., 2013; Yin et al., 2020). To overcome the disadvantages of traditional serotyping, such as the substantial time and labor requirements and the need for large number of specific antisera (Wattiau et al., 2011; Shi et al., 2015), the application of whole-genome sequencing (WGS) for *Salmonella* serotype identification and source tracking is attractive and has been shown to provide accurate results (Allard et al., 2016; Ashton et al., 2016). Two WGS platforms have been used in most previous genomic studies of *Salmonella*, including (1) Illumina (URL: https://www.illumina.com/systems/sequencing-platforms.html), which has been widely used for *Salmonella* identification, source tracking, and surveillance (Allard et al., 2016; Ashton et al., 2016); (2) Oxford Nanopore Technologies (ONT), which provides a solution to sequence long-read nucleic acid fragments in a rapid, real-time manner (URL: https://nanoporetech.com/products). Illumina sequencing generally has a higher data quality than ONT (Fox et al., 2014; Rang et al., 2018), although several studies have demonstrated that ONT sequencing can provide for reliable serotype prediction (Diep et al., 2019; Banerji et al., 2020; Cooper et al., 2020; Xu et al., 2020; Wu et al., 2021). There are reports of completing several *Salmonella* closed genomes using ONT data (González-Escalona et al., 2018; Gao et al., 2020; Haendiges et al., 2021) as well as differentiation of highly similar variants (Xu et al., 2021). These studies used one or two well recognized bioinformatic tools, SISTR (Yoshida et al., 2016) and SeqSero2 (Zhang et al., 2019), for *Salmonella* serotype prediction. Regardless of the data quality, both tools could successfully identify the target *Salmonella* serotype.

WGS data can also be used for the identification of virulence and antimicrobial resistance (AMR) genes. These genes can play critical roles in predicting appropriate treatments and strategies for outbreak control. Initiatives and plans have been formed for proactive monitoring and containment of AMR (USDA, 2014; Smith et al., 2016; WHO, 2016), and these actions would consequently impact food related industries. WGS is currently being used as an effective tool for prediction, surveillance, and further analysis of AMR in different microorganisms (Köser et al., 2014; Oniciuc et al., 2018), such as *Campylobacter jejuni* (Hodges et al., 2021) and *E.coli* (Päivärinta et al., 2020). Compared with a phenotypic antimicrobial susceptibility test, which by definition cannot provide genetic information on the AMR determinants (Kahlmeter et al., 2003), WGS identifies genetic AMR determinants, which has a number of advantages, including more detailed information on AMR emergence. Multiple studies used WGS for genomic analysis and discovered that, as one of the most common food-borne disease-causing bacteria, *Salmonella* isolates from different origins have acquired a large number of AMR and disinfectant resistance genes, with a number of isolates and strains having developed multi-drug resistance (Davidson et al., 2018; Jajere, 2019; Marchello et al., 2020; Zhao et al., 2020; Chen et al., 2020a). As for data acquisition, Chen et al. (2020a) reported that ONT sequencing can provide data for the analysis of AMR genes as well as virulence factors, while a hybrid of ONT and Illumina sequencing data was believed to be a better solution for higher accuracy (Wick et al., 2017; Chen et al., 2020a,b; Neal-McKinney et al., 2021), since it leverages both the high sequencing quality of Illumina data, and the continuity of long-read ONT data. The hybrid method for assembling high quality genomes was introduced as the R9 flow cells have been showing generally lower raw read accuracy compared to Illumina sequencing (Liu et al., 2019). However, with the continuous development of the nanopore sequencing technology, the latest version of the R10 flow cells appears to have improved performance in obtaining high quality raw reads, therefore making it possible to generate near-finished bacterial genomes without Illumina sequencing data for a hybrid assembly (Sereika et al., 2022).

In our previous study, ONT sequencing with multiplexing of five *Salmonella* isolates was evaluated and both SISTR and SeqSero2 results indicated that accurate serotype prediction can be achieved when each multiplexed isolate reached a minimum of 50× genome coverage (Wu et al., 2021). Cross-contamination of barcodes was observed in both our study as well as by Xu et al. (2018), and we suspected the root cause of such cross-contamination was the remaining free adaptors during library preparation and pooling. Removal of the dissociative adaptors would solve the problem. Since only 10 isolates were selected in our previous study, the conclusions above are yet to be evaluated in a larger scale test. Moreover, our previous study only focused on the identification of serotype antigenic formula, neither AMR gene nor virulence gene identification was examined. Consequently, in this study, we aimed to: (1) further evaluate the feasibility and reliability of the five isolate multiplex strategy we proposed before (Wu et al., 2021) using a larger number of isolates; and (2) compare the ability of the ONT and Illumina platforms to identify AMR as well as virulence genes within the genomic data generated.

## 2. Materials and methods

### 2.1. Bacterial strains

Sixty-nine *Salmonella* isolates each representing a different serotype were assessed in the current study (Supplementary Table 1). Thirty-nine of these isolates represented the most common serotypes, which were selected from (i) the most common serotypes reported by the U.S. national *Salmonella* surveillance system in 2016 from all sources (CDC, 2016), (ii) the 20 most frequent serotypes in 2019 in the European Union/European Economic Area (EFSA and ECDC, 2021), and (iii) the most frequently serotyped human *Salmonella* isolates from American, Asian, European, North American, or Oceania countries, based on data extracted from the World Health Organization Global Foodborne Infections Network Country Data Bank (Hendriksen et al., 2011). Five isolates represented rare serotypes found in the food industry (information obtained by personal communication) including serotypes Minnesota, Johannesburg, Cubana, Havana, and Liverpool. Eight isolates represented difficult-to-identify or differentiate serotypes with molecular-level serotyping methods, as described in our previous study (Xu et al., 2020) including serotype Typhimurium, its O5- variant, and serotype 4,[5],12:i:-, Paratyphi B var. Java, Choleraesuis, Virchow, Orion var. 15+, 34+ and Give. Two isolates represented serotypes with issues associated with detection from the food supply chain (information obtained by personal communication), including serotype Poona and 66:z41:- (*S. bongori* subspecies V). The other serotypes not within the above-mentioned categories were randomly selected from the Cornell Food Safety Lab *Salmonella* isolate storage to represent serotypes with relatively moderate prevalence from various sources. Detailed isolate information can be found at www.foodmicrobetracker.com under the isolate ID (e.g., FSL R8-1295).

### 2.2. Genomic DNA extraction

*Salmonella* genomic DNA of all isolates was extracted as previously described (Wu et al., 2021). Briefly, QIAamp DNA mini kit (Qiagen, Hilden, Germany) was applied to extract genomic DNA from single colonies on Trypticase Soy Agar, which were cultured at 37°C for 20 ~ 22 h. Quality of the genomic DNA was assessed with the NanoDrop 2000 (Thermo Fisher Scientific, Delaware, United States) for absorbance value (A value), and the double stranded DNA quantity was assessed with the Qubit 3.0 fluorimeter (Life Technologies, Paisley, United Kingdom). The genomic DNA samples that met the criteria from ONT’s guidance for qualification requirements for successful sequencing were used for library construction: (i) A 260/280 between 1.8 and 1.9; (ii) A 260/230 between 2.0 and 2.2. for each flow cell (FC). For each of the FCs, all multiplexed DNA samples were normalized to the same concentration before input, ranging from 400 to 600 ng. FCs with 1,000–1,500 active pores were used for sequencing.

### 2.3. Oxford nanopore library preparation and sequencing

**Figure 1:**
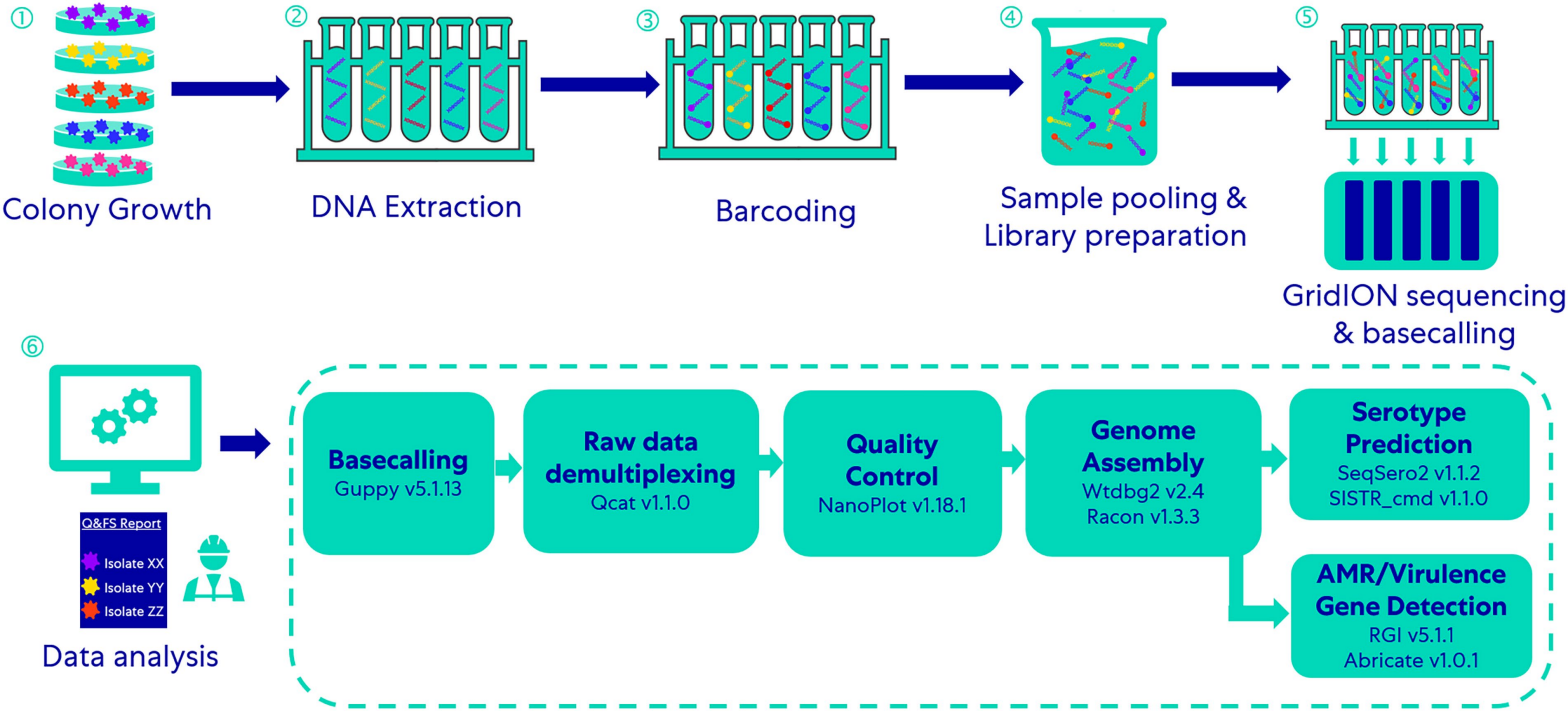
Workflow of the multiplex-ONT-sequencing-based WGS method for *Salmonella* serotype prediction and AMR/virulence gene detection.

The 69 isolates were divided into 14 groups; each group included five different isolates (isolate *Salmonella* Typhi FSL R6-0540 was used in two groups; see Table 1). We multiplexed five DNA libraries from each group into one DNA sample with the rapid Barcoding Sequencing kit (SQK-RBK004) according to the manufacturer’s instructions and sequenced it with qualified FLO-MIN106D FCs (R9.4.1, active pore number ≥ 800) for 24 h on a GridION (Oxford Nanopore Technologies, Oxford, UK; Figure 1). Five barcodes (Barcode 01 ~ 05) were assigned to five isolates in each group and on each FC (Table 1). To assess the capability of this method for differentiating closely related *Salmonella* serotypes, we arranged the serotypes with similar antigenic formulae in the same group. For instance, Group No.1 includes *Salmonella* serotype Typhi (9,12[Vi]:d:-), Barranquilla (16:d:e,n,x), Minnesota (21:b:e,n,x), Gaminara (16:d:1,7), and Johannesburg (1,40:b:e,n,x); two of them hold the same O antigen – “16,” three of them hold the same H1 antigen – “d,” two hold the same H1 antigen – “b,” and three of them hold the same H2 antigen – “e, n, x.” Serotype Typhi does not have an H2 antigen, we thus added it to this group in order to investigate if multiplexed ONT sequencing would lead to a false positive H2 antigen of the serotype Typhi due to possible *in vitro* or *in silico* cross-contamination. We performed basecalling with Guppy’s basecalling model (v5.1.13) integrated in the MinKNOW software v21.11.17 installed on GridION. This model was modified for 6 mA dam/5mC dcm and CpG.

**Table 1:** *Salmonella* isolates tested.

Group ID	Barcode ID	Serotype^1^	Isolate ID (Cornell Food Safety Lab ID)	Antigenic Formula^2^
1	No. 01	Typhi	FSL R6-0540	9,12[Vi]:d:-
1	No. 02	Barranquilla	FSL R8-1295	16:d:e,n,x
1	No. 03	Minnesota	FSL R8-2410	21:b:e,n,x
1	No. 04	Gaminara	FSL R8-5569	16:d:1,7
1	No. 05	Johannesburg	FSL S5-0703	1,40:b:e,n,x
2	No. 01	Mississippi	FSL A4-0633	1,13,23:b:1,5
2	No. 02	Poona	FSL R8-0115	1,13,22:z:1,6
2	No. 03	Cubana	FSL R8-3581	1,13,23:z29:-
2	No. 04	Roodepoort	FSL R8-7983	1,13,22:z10:1,5
2	No. 05	Worthington	FSL S5-0490	1,13,23:z:l,w
3	No. 01	Derby	FSL R8-2630	1,4,[5],12:f,g:[1,2]
3	No. 02	Typhimurium o5-	FSL R8-3714	1,4,[5],12:i:1,2
3	No. 03	Agona	FSL S5-0517	1,4,[5],12:f,g,s:[1,2]
3	No. 04	Typhimurium	FSL S5-0536	1,4,[5],12:i:1,2
3	No. 05	4,[5],12:i:-	FSL S5-0580	4,[5],12:i:-
4	No. 01	Sandiego	FSL R8-4447	1,4,[5],12:e,h:e,n,z15
4	No. 02	Enteritidis	FSL S5-0415	1,9,12:g,m:-
4	No. 03	Paratyphi B var. Java	FSL S5-0447	1,4,[5],12:b:1,2
4	No. 04	Heidelberg	FSL S5-0448	1,4,[5],12:r:1,2
4	No. 05	Saintpaul	FSL S5-0649	1,4,[5],12:e,h:1,2
5	No. 01	Bredeney	FSL R8-2629	1,4,12,27:l,v:1,7
5	No. 02	Kiambu	FSL R8-9562	1,4,12:z:1,5
5	No. 03	Wien	FSL R9-0007	1,4,12,[27]:b:l,w
5	No. 04	*S. enterica* subspecies IIIa -:z4,z23:-	FSL R9-0515	-:z4,z23:-
5	No. 05	Schwarzengrund	FSL S5-0458	1,4,12,27:d:1,7
6	No. 01	Give	FSL S5-0487	3,{10}{15}{15,34}:l,v:1,7
6	No. 02	Orion var. 15+, 34+	FSL R8-3858	3,{10}{15}{15,34}:y:1,5
6	No. 03	Alachua	FSL R8-2924	35:z4,z23:-
6	No. 04	Anatum	FSL R8-7981	3,{10}{15}{15,34}:e,h:1,6
6	No. 05	Muenster	FSL S5-0432	3,{10}{15}{15,34}:e,h:1,5
7	No. 01	*S. bongori* subspecies V 66:z41:-	FSL R9-0518	66:z41:-
7	No. 02	Meleagridis	FSL R8-6670	3,{10}{15}{15,34}:e,h:l,w
7	No. 03	Stockholm	FSL R8-4727	3,{10}{15}:y:z6
7	No. 04	Uganda	FSL R8-3404	3,{10}{15}:l,z13:1,5
7	No. 05	Weltevreden	FSL S5-0438	3,{10}{15}:r:z6
8	No. 01	Choleraesuis	FSL R9-0095	6,7:c:1,5
8	No. 02	Bareilly	FSL R8-7922	6,7,14:y:1,5
8	No. 03	Infantis	FSL S5-0734	6,7,14:r:1,5
8	No. 04	Rissen	FSL R9-0152	6,7,14:f,g:-
8	No. 05	Thompson	FSL S5-0523	6,7,14:k:1,5
9	No. 01	Braenderup	FSL R8-7984	6,7,14:e,h:e,n,z15
9	No. 02	*S. enterica* subspecies IV 45:g,z51:-	FSL R9-0517	45:g,z51:-
9	No. 03	Norwich	FSL R8-6279	6,7:e,h:1,6
9	No. 04	Mbandaka	FSL S5-0451	6,7,14:z10:e,n,z15
9	No. 05	Newport	FSL R8-7979	6,8,20:e,h:1,2
10	No. 01	Havana	FSL S5-0549	1,13,23:f,g,[s]:-
10	No. 02	Livingstone	FSL R8-5215	6,7,14:d:l,w
10	No. 03	Ohio	FSL R8-4333	6,7,14:b:l,w
10	No. 04	Putten	FSL A4-0590	13,23:d:l,w
10	No. 05	Virchow	FSL S5-0961	6,7,14:r:1,2
11	No. 01	Panama	FSL R8-2996	1,9,12:l,v:1,5
11	No. 02	Dublin	FSL S5-0439	1,9,12[Vi]:g,p:-
11	No. 03	Ibadan	FSL R8-4726	13,22:b:1,5
11	No. 04	Javiana	FSL S5-0395	1,9,12:l,z28:1,5
11	No. 05	Pomona	FSL R8-0451	28:y:1,7
12	No. 01	*S. enterica* subspecies VI [1],6,14,[25]:a:e,n,x	FSL R9-8566	[1],6,14,[25]:a:e,n,x
12	No. 02	Cerro	FSL R8-0370	6,14,18:z4,z23:[1,5]
12	No. 03	Senftenberg	FSL R8-5370	1,3,19:g,[s],t:-
12	No. 04	Hartford	FSL R8-5223	6,7:y:e,n,x
12	No. 05	*S. enterica* subspecies IIIb 6,7:l,v:z53	FSL R9-0516	6,7:l,v:z53
13	No. 01	Kentucky	FSL S5-0273	8,20:i:z6
13	No. 02	Typhi^3^	FSL R6-0540	9,12[Vi]:d:-
13	No. 03	Blockley	FSL S5-0648	6,8:k:1,5
13	No. 04	Muenchen	FSL R8-7982	6,8:d:1,2
13	No. 05	Apapa	FSL R8-5222	45:m,t:-
14	No. 01	Liverpool	FSL R9-1184	1,3,19:d:e,n,z15
14	No. 02	Oranienburg	FSL R8-7977	6,7,14:m,t:[z57]
14	No. 03	Ealing	FSL R8-2454	35:g,m,s:-
14	No. 04	Montevideo	FSL S5-0630	6,7,14,[54]:g,m,[p],s:[1,2,7]
14	No. 05	Tennessee	FSL R8-5221	6,7,14:z29:[1,2,7]

1 Serotype information of each isolate was based on data from www.foodmicrobetracker.com under the isolate ID.

2 Antigenic formula was extracted from Grimont and Weill (2007). Antigenic formulae of the *Salmonella serovars*, (9th ed.) Paris: WHO Collaborating Centre for Reference and Research on *Salmonella*, according to the serotype names.

3 Isolate FSL R6-0540 (Serotype Typhi) was used in two groups - Group 1 and Group 13.

### 2.4. Genomic and serotype prediction analysis

Raw data (demultiplexed, unfiltered and untrimmed reads) obtained after basecalling were processed through essentially the same demultiplexing (qcat v1.1.0, https://github.com/nanoporetech/qcat) and genome assembling workflow described by Wu et al. (2021); Figure 1. We used NanoPlot (version 1.18.1) to analyze the quality of ONT raw sequencing data.

Original serotype information for isolates was received by the source that provided isolates; our understanding is that all serotype data for the isolates used here was based on classical, antibody-based, serotyping (and not based on serotype prediction based on molecular data, e.g., WGS data). A large proportion of the isolates was obtained from animals and humans; these isolates had typically been characterized by traditional serotyping performed by agglutination (as described by Edwards and Ewing, 1986) at either the New York State Department of Health (for human isolates) or the National Veterinary Services Laboratories (NVSL), a division of the United State Department of Agriculture (USDA) Animal and Plant Health Inspection Service (APHIS, Ames, Iowa; for animal isolates; Alcaine et al., 2006; Rodriguez-Rivera et al., 2014). Both SeqSero2 v1.1.2^1^ and SISTR_cmd (The *Salmonella in silico* Typing Resource Command-line Tool) v1.1.0 (Yoshida et al., 2016) were used for serotype prediction with the sequence data generated in this study. As previously described (Wu et al., 2021), both ONT raw reads and assembled contigs were used as input data for SeqSero2, while only assembled contigs were used as input data for SISTR. Default parameters were used according to the developer’s manual. We collected different sizes of sequencing data from ONT to assess the influence of sequencing depth and sequencing time on the accuracy of serotype prediction. We defined the lowest depth of genome coverage that one multiplexed isolate could achieve among all the multiplexed isolates on one FC at a given sequencing time as Depth_min_ of this FC. A one-way analysis of variance (ANOVA) followed by a Tukey HSD test was carried out to compare the difference of data yield between combined isolates in each FC.

### 2.5. AMR and virulence gene identification and precision-recall analysis

Assembled contigs generated from our serotype prediction workflow were used for AMR and virulence gene identification. RGI (resistant gene identifier) 5.1.1 (Alcock et al., 2020) and Abricate 1.0.1^2^ were both loaded with the CARD database (ver. 2020-Apr-19; Alcock et al., 2020) and then launched for the identification of AMR genes under default arguments. For virulence gene identification, only Abricate was used to search against the VFDB database (ver. 2020-Apr-19; Liu et al., 2019), and arguments were defined as default.

A precision-recall analysis was performed to evaluate the differences between Illumina and ONT data regarding the AMR and virulence genes identified. AMR and virulence genes identified from Illumina data were assumed to be the benchmark. Therefore, for each isolate, genes identified in both Illumina and ONT data were true positive (TP) results, genes identified only in ONT data were false positive (FP) results, and genes identified only in Illumina data were false negative (FN) results. The following equations were used to obtain the values of “Precision,” “Recall,” “Accuracy,” and “False-negative probability”: False-positive probability was not calculated as we did not pursue the true negative number of AMR or virulence genes detected in this study.


Precision=∑TP/∑(TP+FP)

Recall=∑TP/∑(TP+FN)

Accuracy=∑(TP+TN)/∑(TP+TN+FP+FN)

$$\begin{array}{c} \text{False}-\text{negative}\\ \text{probability} \end{array}=\;\begin{array}{c} \sum \text{False negative}/\\ \;\;\;\sum \left(\text{False negative}+\text{True Positive}\right) \end{array}\;$$

To investigate the impact of depth of genome coverage on Precision and Recall, different depths (15×, 30×, 50×, and 75×) of genome coverage data for the serotype Typhimurium isolate were extracted to perform genome assembling, the obtained contigs were analyzed through AMR and virulence gene identification by RGI and Precision-Recall analysis.

### 2.6. Identification of possible cross-assigned reads and influence of cross-assigned reads on the accuracy of serotype prediction

We detected possible cross-assigned reads from each FC as described previously (Wu et al., 2021). Cross-assigned reads were identified from serotype prediction errors caused by single ONT reads. We used 50× depth of genome coverage demultiplexed raw sequencing reads as input of SeqSero2 to identify this type of prediction error and the corresponding error-causing antigen determinant loci. ONT raw reads that could match these error-causing antigen determinant loci, by using BLAST^3^ with identity ≥90% and coverage = 100%, would be classified as possible cross-assigned reads.

## 3. Results and discussion

### 3.1. Overview of multiplexed and demultiplexed nanopore sequencing data and assembly of *Salmonella* genomes

An average of 6.58 Gbp of raw ONT sequencing data per FC (*N* = 14 FCs) was obtained after 24 h of ONT sequencing. Data outputs of FCs ranged from 4.51 to 8.34 Gbp with a mean read length of 9,248 bp and a mean N50 read length of 17,273 bp on average across all FCs (Table 2). Sequencing quality was shown to be highly consistent among FCs, with mean quality scores for a given FC ranging from 11.50 to 12.10. The average quality score across all FCs was 11.79.

**Table 2:** Statistics of ONT multiplex sequencing data for each group of isolates tested from 24 h of sequencing.

Group No^1^.	Total clean data yield in 24 h (Gbp)	Mean read length (bp)	Mean quality score	Number of reads	Mean read length N50
1	6.70	8,527	11.50	786,309	17,240
2	6.17	8,842	11.70	697,460	16,854
3	7.19	8,240	12.10	872,570	15,974
4	6.68	8,747	11.20	873,088	17,622
5	7.19	8,772	11.50	819,834	16,281
6	5.31	9,253	12.10	573,709	17,183
7	5.90	9,205	11.80	640,597	16,709
8	7.64	9,629	11.90	693,913	17,290
9	5.78	8,431	12.00	685,709	15,234
10	7.20	10,475	12.00	687,001	19,509
11	8.34	8,486	11.90	982,440	15,686
12	5.68	11,081	11.90	512,726	19,932
13	7.79	10,304	11.70	756,070	18,957
14	4.51	9,475	11.70	476,383	17,348
**Average**	**6.58**	**9,248**	**11.79**	**718,415**	**17,273**

1 Each group contains five isolates, each group of isolates were multiplexed and sequenced on one ONT flow cell.

**Figure 2:**
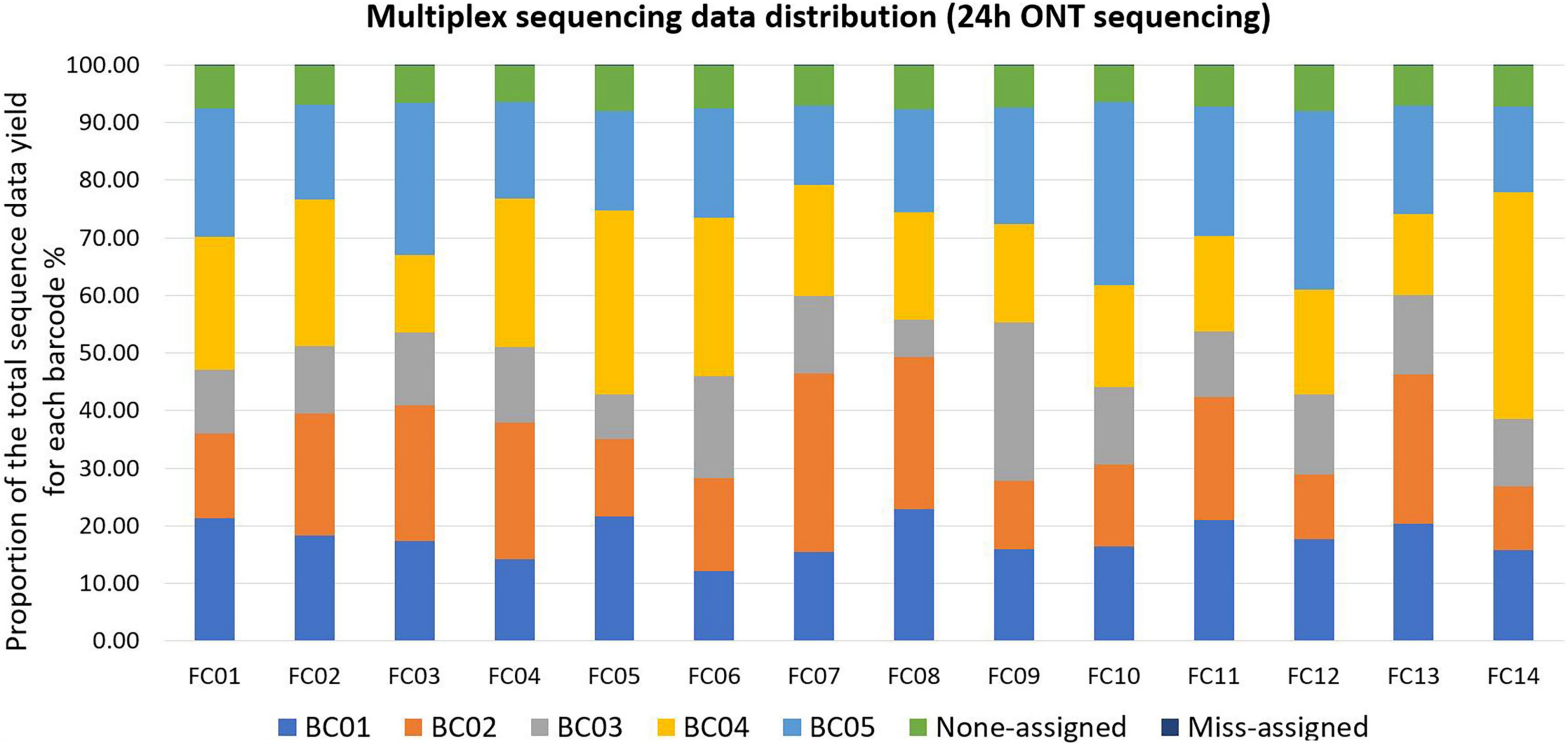
Multiplex sequencing data distribution within each flow cell (FC) for each barcode (BC) after 24 h of ONT sequencing. Each color represents one BC or none-assigned/miss-assigned reads. Within each FC, ONT reads that were not assigned to any BC were defined as non-assigned reads, reads assigned to a BC that was not used in the FC were defined as miss-assigned reads. As the bars for miss-assigned reads were not easily visible, detailed proportions are listed here: the number of mis-assigned reads was 2% in FC02 and FC05, and 3% in all the other FCs.

Qcat failed to assign an average of 7.20% ± 0.52% (mean ± standard deviation, *N* = 14 FCs) reads per FC to any barcode; these reads were defined as Non-assigned reads. Qcat assigned an average of 0.03% reads per FC to barcodes that were not included in the FC, which was consistent with our previous study (Wu et al., 2021). These reads were defined as mis-assigned reads (Figure 2; Supplementary Table 2). The original demultiplexed ONT sequencing data were submitted to NCBI - SRA (Accession number: PRJNA694442).

**Figure 3:**
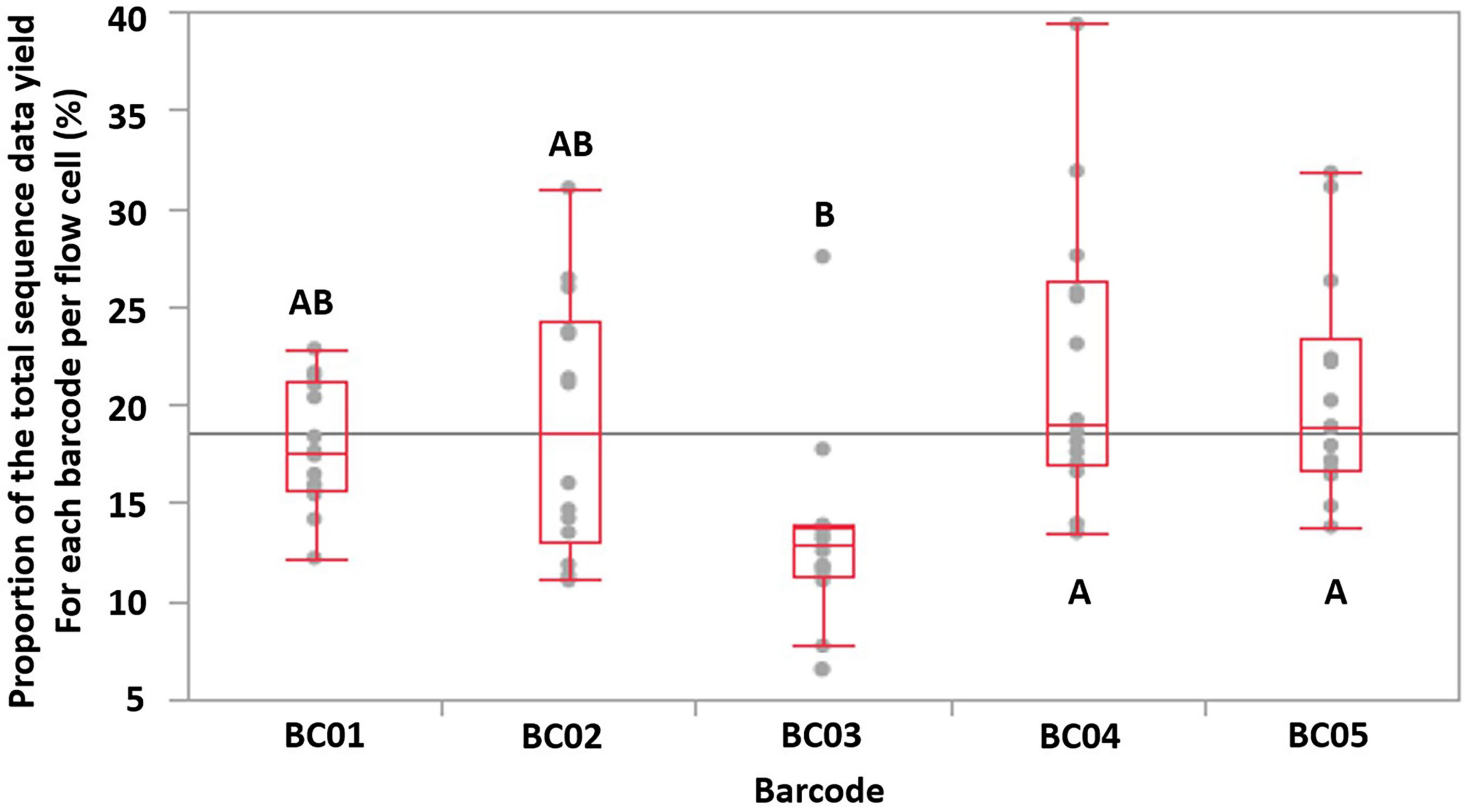
The proportion of the total sequence data yield for each barcode (labeled as BC01-BC05) per flow cell is shown as a single dot. The maximum/minimum (excluding outliers), 75, 50, and 25% quantile data yield of a barcode of each flow cell are labeled with red lines. Based on Tukey HSD test, barcodes that does not share the same letters were significantly different (*p*-value < 0.05, overall ANOVA *p*-value = 0.0019).

Tukey HSD test indicated that Barcode 03 (BC03) showed significantly lower (*p* < 0.05, *N* = 14 FCs, overall ANOVA *p*-value = 0.0019, α = 0.05) sequence data yields compared to two of the other four barcodes used, which implied that the data-yield performance varied among the barcodes provided by the same ONT rapid barcoding kit product (Figure 3). This may lead to uneven distribution of sequence data among multiplexed isolates on the same FC, impacting the minimum total ONT sequencing time, because the final total sequencing time needs to be long enough to allow the barcoded isolate that obtained the least sequence data to receive sufficient data (e.g., 50× depth of genome coverage) for downstream analysis. Our previous study also identified that, when multiplexing more than four isolates, some of the barcodes showed significantly lower data yield than the others (Wu et al., 2021).

### 3.2. Influence of sequencing time and depth on accuracy of *Salmonella* serotype prediction using ONT assembled genomes

With five isolates multiplexed on each FC, we assessed three levels of Depth_min_ (15×, 30×, 50×) for the accuracy of *Salmonella* serotype prediction. Using assembled genomes as input to SeqSero2 and SISTR, when Depth_min_ was 30× and 50×, SeqSero2 correctly predicted all 69 serotypes across 14 FCs, while the prediction accuracy of SISTR was 98.6% (68/69) at Depth_min_ 30× and 50× of the FC (Table 3). The error result generated by SISTR showed that the O antigen of serotype -:z4,z23:- (subspecies IIIa, FSL R9-0515) was miss-called as “41” across all the depths tested (Table 4). The O antigen of the other four isolates multiplexed with FSL R9-0515 are all “4” rather than “41,” and correctly predicted, indicating that this prediction error was not caused by cross-assigned reads. Cross-assigned reads analysis by BLAST did not identify any cross-assigned read that could cause the miss-calling of O antigen as “41.”

**Table 3:** Accuracy of *Salmonella* serotype prediction using assembled genomes from ONT multiplex sequencing data.

Depth_min_^1^	SeqSero2	SISTR
15 x	95.7% (66/69)	97.1% (67/69)
30 x	100% (69/69)	98.6% (68/69)
50 x	100% (69/69)	98.6% (68/69)

1 We defined the lowest depth of genome coverage that one multiplexed isolate could achieve among all the multiplexed isolates on one flow cell at a given sequencing time as Depthmin of this flow cell.

**Table 4:** *Salmonella* serotype prediction errors.

Isolate ID	Serotype	Antigenic Formula	Predicted Antigenic Formula	Serotype prediction tool	Actual depth of genome coverage	Depth_min_ of the flow cell
FSL R8-1295	Barranquilla	16:d:e,n,x	I -:d:e,n,x	SeqSero2	22×	15×
FSL R8-2410	Minnesota	21:b:e,n,x	I -:b:e,n,x	SeqSero2	16×	15×
FSL R9-0517	IV 45:g,z51:-	45:g,z51:-	IV -:g,z51:-	SeqSero2	17×	15×
FSL S5-0648	Blockley	6,8:k:1,5	I -:k:1,5	SeqSero2	17×	15×
FSL R8-5370	Senftenberg	1,3,19:g,[s],t:-	3,10:g,s,t:-	SeqSero2	20×	15×
FSL R9-0515	subspecies:IIIa -:z4,z23:-	-:z4,z23:-	IIIa 41:z4,z23:-	SISTR	60×; 122×; 206×	15×; 30×; 50×

With respect to the prediction errors at Depth_min_ 15× of SeqSero2 and SISTR, BLAST analysis showed that the low depth of genome coverage (15×) with the relatively low sequencing quality of ONT data (average Qscore = 12.24 at Depth_min_ of 15×, *N* = 14) led to failure in matching antigen determinant alleles of O antigen 1, 3, 6, 16, 19, 21, or 45, for five serotypes. For all the prediction errors of SeqSero2 at Depth_min_ of 15× (Table 4), the low depth of coverage did not allow SeqSero2 to determine any O antigen for four of the tested serotypes.

The ONT sequencing times for each Depth_min_ level are shown below (Table 5). Depth_min_ 15×, 30×, and 50× of one FC multiplexing five *Salmonella* isolates could be achieved within 2.36 ± 0.43 h, 4.32 ± 0.87 h, and 7.01 ± 1.57 h (mean ± standard deviation, N = 14), respectively.

**Table 5:** ONT multiplex sequencing time per flow cell.

Group ID	Sequencing Time in hours to achieve a minimum genome coverage for each isolate on a flow cell		
	15×	30×	50×
Group 1	2.80	4.96	7.67
Group 2	2.30	4.14	6.72
Group 3	1.94	3.49	5.52
Group 4	1.87	3.48	5.67
Group 5	2.66	5.12	8.60
Group 6	2.37	4.38	7.23
Group 7	1.98	3.51	5.65
Group 8	3.24	6.19	10.45
Group 9	2.38	4.32	6.98
Group 10	1.90	3.41	5.37
Group 11	2.17	3.76	5.82
Group 12	2.86	5.27	8.34
Group 13	1.97	3.47	5.41
Group 14	2.61	4.97	8.64
Average Sequencing Time (*N* = 14)	2.36	4.32	7.01

In summary, accurate serotype prediction with ONT WGS data was achieved within about 5 h of ONT sequencing at a minimum depth of *Salmonella* genome coverage at 30× (assuming the genome size of a given *Salmonella* strain is 4.8 Mbp) with Guppy’s basecalling model modified for 6 mA dam/5mC dcm and CpG, with SeqSero2 as the prediction tool. This minimum depth of ONT WGS data for serotype prediction is also consistent the minimum depth (for ≥10 kb reads) for bacterial assemblies recommended by ONT (URL: https://nanoporetech.com/sites/default/files/s3/literature/microbial-genome-assembly-workflow.pdf).

As we previously reported, multiplexing more than three isolates will inevitably cause uneven data allocation among multiplexed isolates. It is thus essential that the multiplexed isolate with the least data reaches a depth of 30× genome coverage for reliable serotype prediction, by extending overall sequencing duration to be longer than simply using the sequencing time required for a single isolate for a multiplexed run.

When using raw reads rather than assembled genomes as input for SeqSero2, one serotype prediction error was found for isolate FSL R9-0518 (*Salmonella Bongori*; Serotype 66:z41:-) with FC Depth_min_ at 50×, where it was called as *Salmonella Bongori* Serotype 1,3,19:z41:l,w.. Cross-assigned reads analysis by BLAST indicated that the O and H2 antigen were mis-identified, possibly due to reads cross-assigned from the other isolates multiplexed in the same FC. These cross-assigned reads contained antigen determining alleles from the other isolates with O and H2 antigens different from serotype 66:z41:-. With a bead clean-up step added to the multiplexed library preparation process, we found an occurrence rate of read cross-assignment of 7% (1/14) in the current study, compared to 8% (2/24) reported in our previous study (Wu et al., 2021). The errors caused by cross-assigned reads were corrected by using an assembled genome, and this finding is consistent with our previous study (Wu et al., 2021).

### 3.3. AMR and virulence gene identification

At the single isolate level, some variations in AMR and virulence gene profile were observed between Illumina and ONT data (Supplementary Table 3). For an individual *Salmonella* isolate, Abricate generated similar AMR and virulence identification results using either Illumina or ONT data, while RGI showed substantial discrepancies between these two sequencing platforms. Taking results from Illumina data as ground truth, the average number of true positive AMR genes per isolate identified from ONT data by Abricate and RGI was 26.19 and 29.64, respectively (Table 6). With ONT data, Abricate and RGI (i) failed to identify an average of 0.36 and 11.81 AMR genes per isolate, respectively and (ii) identified an average of 0.19 and 5.91 false positive AMR genes per isolate, with false negative probabilities of 1.37 and 27.62%, respectively. When using Abricate with the VFDB library to scan ONT data for virulence genes per isolate, 99.64 true positive virulence genes on average were identified, with 1.25 false negative genes and 1.07 false positive genes identified. In general, if RGI was used for AMR gene identification, ONT data of the 69 isolates yielded an average precision of 0.84 and an average recall of 0.72 compared to Illumina data. Abricate on the other hand yielded both precision and recall of 0.99 for either AMR gene or virulence gene identification.

**Table 6:** Accuracy of AMR/virulence gene identification using ONT multiplex sequencing data.

	RGI (CARD)		Abricate (CARD)		Abricate (VFDB)	
	Average Value^2^	95% CI	Average Value	95% CI	Average Value	95% CI
True positive^1^	29.64	28.92, 30.36	26.19	25.64, 26.74	99.64	97.77, 101.51
False negative	11.81	10.49, 13.13	0.36	0.16, 0.56	1.25	0.56, 1.94
False positive	5.91	5.45, 6.37	0.19	0.06, 0.32	1.07	0.43, 1.71
Precision^3^	0.84	0.83, 0.85	0.99	0.99, 0.99	0.99	0.98, 1.00
Recall^4^	0.72	0.70, 0.74	0.99	0.98, 1.00	0.99	0.98, 1.00
False-negative probability^5^	27.62%	25.44, 29.80%	1.37%	0.61, 2.13%	1.18%	0.54, 1.82%

1 The positive detections of AMR or virulence genes from Illumina sequence data were taken as true positives; any genes that were detected by Illumina sequence data, but not by ONT sequence data were taken as false negatives; any genes were detected by ONT sequence data, but not by Illumina sequence data were taken as false positives.

2 N = 69 *Salmonella* isolates.

3 Precision = ∑True positive/∑(True positive + False positive).

4 Recall = ∑True positive/∑(True positive + False negative).

5 False-negative probability = ∑False negative/ ∑(False negative + True Positive); False-positive probability was not calculated as we did not pursue the true negative number of AMR or virulence genes detected in this study.

**Figure 4:**
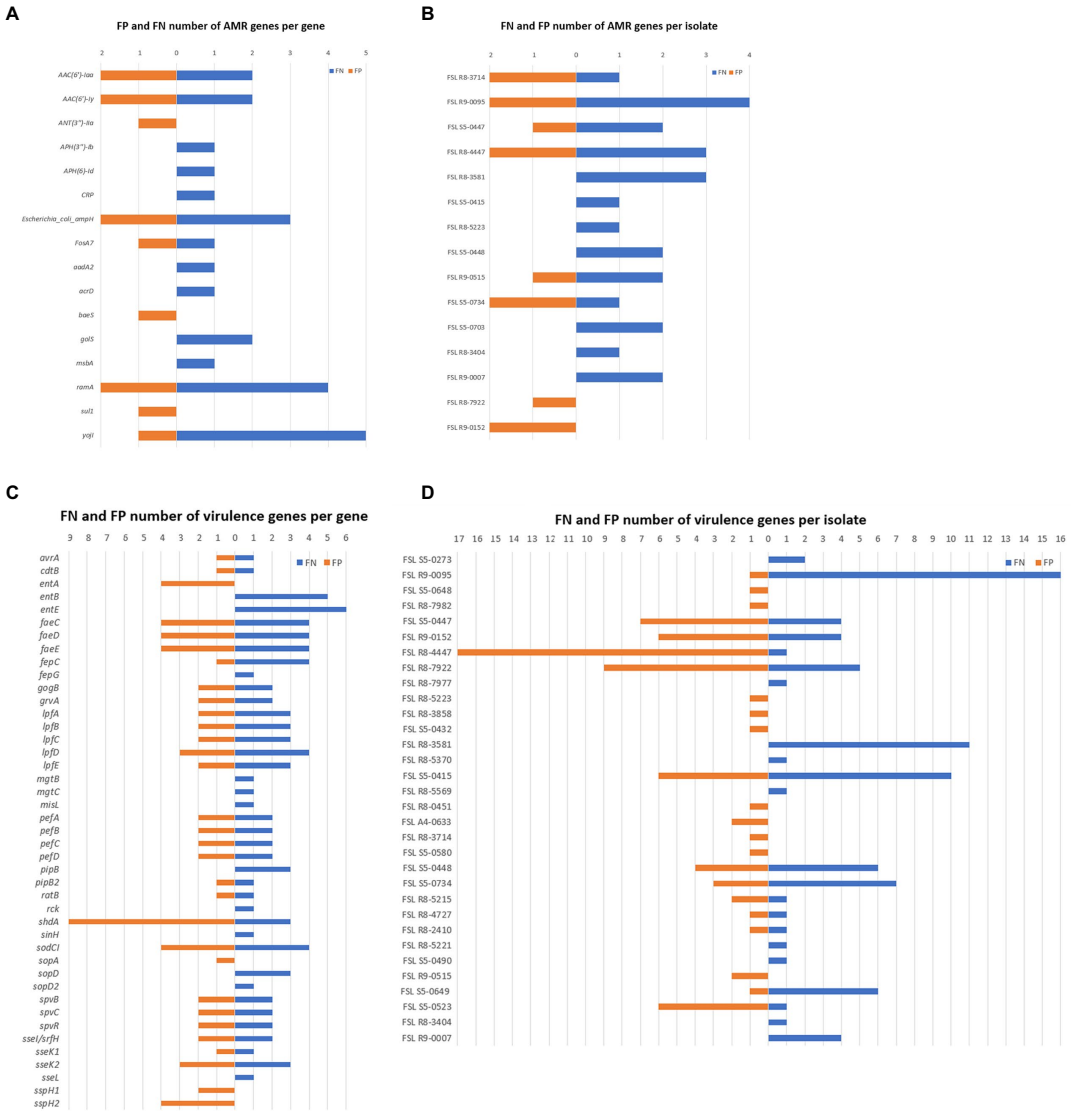
False Positive (FP) and False Negative (FN) numbers of AMR and virulence genes detected with ONT data, taking results from Illumina data as benchmark. **(A)** Number of AMR gene FN (in orange) and FP (in blue) results for different AMR genes; AMR genes not shown did not yield AMR gene FP or FN results; **(B)** Number of AMR gene FN and FP results for each given isolate; isolates not shown did not yield AMR gene FN or FP results; **(C)** Number of virulence gene FN and FP results for different virulence genes; virulence genes not shown did not yield virulence gene FP or FN results; **(D)** Number of virulence FN and FP results for each given isolate; isolates not shown did not yield virulence gene FN or FP results.

Through the results generated by Abricate, we calculated the total number of false negatives (FNs) and false positives (FPs) for each detected gene as well as for each isolate. A total of 26 isolates were found as having FNs; these FNs were associated with a total of 13 AMR and 39 virulence genes (Figure 4), indicating these genes failed to be detected in at least some of the genome assemblies generated from ONT sequencing data. The raw reads containing these FNs might have been excluded due to low quality caused by ONT sequencing errors, while Illumina sequencing data seemed to provide a more complete profile of the AMR and virulence genes. On the other hand, FPs were associated with nine AMR genes and 31 virulence genes; these FNs were contributed by 23 isolates. The total number of FNs was higher than FPs, although many of the isolates had both FNs and FPs. Among all FPs, one virulence gene *shdA* has an exceptionally high number of FP hits from nine different isolates (Figure 4; Supplementary Table 3), with a mean coverage >97.95% and a mean identity >94.33%. *shdA* encodes an AIDA (Adhesin Involved in Diffuse Adherence)-like protein, with a total nucleotide length of 6,105 bp (Kingsley et al., 2000); such length can be fully covered by ONT long-reads while several Illumina reads are needed for assembly. The FP hits for *shdA* may represent true positives as the true composition of AMR genes and virulence genes of the tested isolates was unknown, because only the Illumina sequencing data were used as the benchmark. It is possible that some genes were not fully recovered during the Illumina-based genome assembly process, consequently causing low coverage or identification when detecting AMR and virulence genes using Abricate.

We compared the precision and recall of AMR/virulence gene identification between five different sequencing depths of one test isolate (FSL S5-0536) representing serotype Typhimurium to further explore the impact of sequencing depth on accuracy of AMR/virulence gene identification with multiplex-ONT WGS data. As all the isolates were tested with the same workflow, we speculated that the dynamics of the association between accuracy and sequencing depths for AMR/virulence gene identification with ONT data would be the same for these isolates. We therefore picked only serotype Typhimurium as the representative and assembled the genome of this isolate at sequencing depth 15×, 30×, 50×, 75×, and 100×. These assemblies then went through Abricate loaded with CARD and VFDB libraries and RGI loaded with CARD library. Recall and Precision statistics based on the identification results are shown below (Table 7). We found that with Abricate, sequencing depth of 30× or above was sufficient to obtain highest recall and precision for both AMR and virulence gene identification for isolate FSL S5-0536, while with RGI, precision and recall reached the highest value when sequencing depth was at 75×.

**Table 7:** Recall and precision of AMR/virulence gene identification for *Salmonella* serotype Typhimurium using assemblies from WGS data at different sequencing depths.

Parameters	Sequencing Depth (*Salmonella* genome size 4.8Mbp)				
	15×	30×	50×	75×	100×
Abricate + CARD (AMR gene identification)					
Recall	96%	100%	100%	100%	100%
Precision	100%	100%	100%	100%	100%
Abricate + VFDB (Virulence gene identification)					
Recall	100%	100%	100%	100%	100%
Precision	100%	100%	100%	100%	100%
RGI + CARD (AMR gene identification)					
Recall	68%	82%	81%	84%	84%
Precision	76%	86%	84%	84%	84%

Previously, Gargis et al. (2019) also found that AMR markers could be correctly detected from the biothreat pathogens *Bacillus anthracis* and *Yersinia pestis* with 100,000 ONT raw sequencing reads per isolate. Noting that *Bacillus anthracis* and *Yersinia pestis* have similar genome sizes as *Salmonella* (assuming the genome size of a given *Salmonella* strain is 4.8 Mbp), our sequencing workflow could usually achieve an accurate AMR profile prediction with around 20,000 raw sequencing reads per isolate (at around 30 × coverage) using Abricate. The differences in precision and recall between RGI and Abricate were likely at least partially due to different default similarity cut-off values for sequence alignment through BLAST used with both softwares. In RGI (Alcock et al., 2020), sequence matching hits are classified into three types: Perfect, Strict, or Loose. Except for the 100% match as “Perfect,” “Strict,” or “Loose” hits will include hits with much lower identity or coverage. RGI generated results from all three types of sequence matching hits, this may have led to some inaccurate identification of AMR with “Strict” or “Loose” hits. In this study, more AMR genes were identified using RGI than Abricate, the least identity in RGI results was 51.35% with a gene coverage of 91.8%, whereas the Abricate results only included hits with minimum identity and coverage of 80 and 90%, respectively. Higher identity and coverage of sequence matching hits adopted by Abricate compared to RGI may increase the accuracy of AMR identification by Abricate when using ONT data. A more recent study (Tan et al., 2020) found that AMR profiles of *Streptococcus suis* could be identified for 100% of the 10 isolates tested using RGI (with CARD library) with ONT sequencing data generated by a MinION sequencer. “Loose” algorithm of RGI was also used for MinION assemblies in Tan et al.’s study, while our findings implied that some of the AMR genes identified by “Loose” algorithm of RGI from ONT sequencing data may not be accurate as compared to results generated by Illumina data for *Salmonella* when coverage is from 15× to 100×.

### 3.4. Recommendation for cost-effective multiplexing strategy for serotype prediction and AMR/virulence gene prediction

The current study shows that, when multiplexing five *Salmonella* genome DNA samples in one ONT FC, 50× or greater depth of genome coverage per isolate/sample allows for accurate serotype prediction and AMR/virulence gene profiling with accuracy comparable to Illumina data for *Salmonella*. The sequencing time for obtaining at least 50× per multiplexed isolate was 7.01 h on average (range: 5.37 to 10.45 h) based on data from sequencing of 14 FCs with five different serotypes multiplexed in each FC, and 69 different *Salmonella* serotypes tested in total. A cost estimation of ONT sequencing with five *Salmonella* isolates multiplexed in one FC has been made in our previous study (Wu et al., 2021). These recommendations are based on the ONT GridION sequencer, FC R9.4.1, sequencing kit SQK-RBK004, basecalling model modified for 6 mA dam/5mC dcm and CpG, as well as the corresponding bioinformatics pipeline developed for the current study. As various sequencing platforms, sequencing kits and bioinformatics tools are available for ONT sequencing, any deviation from or further improvement of the factors described above may change the prediction results and accuracy significantly. Although the sequencing kit (SQK-RBK004) used in this study has the capability of barcoding up to 12 different isolates in one FC, we focused on validating only five isolates multiplexed as we have demonstrated in our previous study that multiplexing five *Salmonella* genome DNA isolates could achieve the most efficient combination of sequencing time, data distribution stability, and cost reduction. Previously, we have demonstrated that the unevenness of data yield between each multiplexed isolate increases significantly as the multiplexing number of isolates increases; and multiplexing seven to 10 isolates resulted in only a small cost benefit, considering their much longer sequencing time (more than 19 h; Wu et al., 2021).

Abricate combined with the CARD/VFDB library is recommended for *Salmonella* AMR/virulence gene identification, as it showed relatively higher accuracy compared to RGI at 50× genome coverage for AMR identification. For AMR/virulence gene detection with ONT sequence data, the choice of appropriate algorithm and setting is particularly important to reduce the impact of relatively low sequencing accuracy of ONT data compared to Illumina data. And to maintain the advantage of the fast turnaround time of ONT sequencing. Meanwhile, a combination of SISTR and SeqSero2 results are recommended for predicting serotypes of *Salmonella*. Although SISTR had slightly lower accuracy compared to SeqSero2 in the current study, they each have advantages in different aspects as they use different algorithms and databases for serotype prediction (Yoshida et al., 2016; Zhang et al., 2019; Xu et al., 2020; Wu et al., 2021). Although it is unknown if certain strains of *Salmonella* would alter the accuracy of serotyping under the recommended settings, the 69 serotypes we covered in the current study represented the majority of common serotypes and major types of H and O antigens of *Salmonella*. Further validation and verification with more *Salmonella* serotypes can be carried out during practice, for example in the food industry or in public health.

## 4. Conclusion

In this study we evaluated the ONT-multiplex-sequencing-based WGS method for *Salmonella* serotype prediction and AMR/virulence gene detection, using Illumina sequencing data for bench marking. We demonstrated that for all the 69 *Salmonella* serotypes tested, accurate serotype prediction and AMR/virulence gene profiling can be obtained with an average of 7 h of ONT sequencing when multiplexing five *Salmonella* serotypes. The accuracy was comparable to results from Illumina data. Multiplexing five isolates results in a 23% reduction to the cost of ONT sequencing of a single isolate per FC. Meanwhile, the workflow we developed also allows for *Salmonella* serotype prediction and AMR/virulence gene detection to be completed within one working day. This study is an evaluation of multiplex-nanopore-sequencing based WGS as a cost-effective and rapid *Salmonella* classification method. It is also a starting point for exploring the application of ONT-based WGS in AMR and virulence gene detection for the food safety area. Our findings pave the way for the application and standardization of ONT-based WGS in surveillance, tracking, and risk assessment of *Salmonella* across the food supply chain as a cost-effective and rapid *Salmonella* classification and AMR/virulence gene profiling tool.

## Data availability statement

The original contributions presented in the study are publicly available. This data can be found here: NCBI, PRJNA694442.

## Author contributions

ST, CG, XW, MW, RB, AS, and GZ contributed to conception and design of the study. XW and HL organized the database. ST performed the statistical analysis. XW, ST, and HL wrote the first draft of the manuscript. XD and FX contributed to sections of the manuscript. All authors contributed to the article and approved the submitted version.

## Funding

This work was funded by Mars Global Food Safety Center, Mars, Incorporated. The funder was not involved in the study design, collection, analysis, interpretation of data, the writing of this article, or the decision to submit it for publication.

## Conflict of interest

XW, HL, CG, FX, RB, AS, GZ, and ST were employed by Mars, Incorporated. MW received compensation to serve as consultant to Mars.

The remaining author declares that the research was conducted in the absence of any commercial or financial relationships that could be construed as a potential conflict of interest.

## Publisher’s note

All claims expressed in this article are solely those of the authors and do not necessarily represent those of their affiliated organizations, or those of the publisher, the editors and the reviewers. Any product that may be evaluated in this article, or claim that may be made by its manufacturer, is not guaranteed or endorsed by the publisher.

## Footnotes

^1^ https://github.com/denglab/SeqSero2

^2^ https://github.com/tseemann/abricate

^3^ https://blast.ncbi.nlm.nih.gov/Blast.cgi
