# Supplementary material for: Evaluation of multiplex nanopore sequencing for Salmonella serotype prediction and antimicrobial resistance gene and virulence gene detection
Source: Front Microbiol. 2023 Feb 1;13:1073057. doi: 10.3389/fmicb.2022.1073057 (PMC9930645; doi:10.3389/fmicb.2022.1073057)
Supplement: Supplementary file 2 [file Data_Sheet_1.docx]

**Supplementary Table 1**. Sixty-nine *Salmonella* isolates tested, each representing a different serotype

| No. | Serotype | Isolate ID  (Cornell Food Safety Lab ID) | Isolation source | Category | Most common | Difficult to predict the serotype correctly | Difficult to differentiate serotypes (genetically similar) | Issues with detection serotypes | Rare in the food industry |
| --- | --- | --- | --- | --- | --- | --- | --- | --- | --- |
| 1 | Bredeney | FSL R8-2629 | NA | 1 | Yes |  |  |  |  |
| 2 | Sandiego | FSL R8-4447 | human, clinical | 1 | Yes |  |  |  |  |
| 3 | Livingstone | FSL R8-5215 | human, clinical | 1 | Yes |  |  |  |  |
| 4 | Tennessee | FSL R8-5221 | NA | 1 | Yes |  |  |  |  |
| 5 | Senftenberg | FSL R8-5370 | NA | 1 | Yes |  |  |  |  |
| 6 | Bareilly | FSL R8-7922 | NA | 1 | Yes |  |  |  |  |
| 7 | Wien | FSL R9-0007 | NA | 1 | Yes |  |  |  |  |
| 8 | Choleraesuis | FSL R9-0095 | human, clinical | 1 | Yes | Yes |  |  |  |
| 9 | Rissen | FSL R9-0152 | human, clinical | 1 | Yes |  |  |  |  |
| 10 | Muenster | FSL S5-0432 | bovine | 1 | Yes |  |  |  |  |
| 11 | Weltevreden | FSL S5-0438 | human | 1 | Yes |  |  |  |  |
| 12 | Schwarzengrund | FSL S5-0458 | human | 1 | Yes |  |  |  |  |
| 13 | Worthington | FSL S5-0490 | human | 1 | Yes |  |  |  |  |
| 14 | Blockley | FSL S5-0648 | human, clinical | 1 | Yes |  |  |  |  |
| 15 | Mississippi | FSL A4-0633 | human | 2 | Yes |  |  |  |  |
| 16 | Cerro | FSL R8-0370 | animal, non-clinical | 2 | Yes |  |  |  |  |
| 17 | Panama | FSL R8-2996 | human, clinical | 2 | Yes |  |  |  |  |
| 18 | Oranienburg | FSL R8-7977 | NA | 2 | Yes |  |  |  |  |
| 19 | Kentucky | FSL S5-0273 | bovine | 2 | Yes |  |  |  |  |
| 20 | Dublin | FSL S5-0439 | human | 2 | Yes |  |  |  |  |
| 21 | Muenchen | FSL R8-7982 | NA | 3 | Yes |  |  |  |  |
| 22 | Javiana | FSL S5-0395 | human | 3 | Yes |  |  |  |  |
| 23 | Anatum | FSL R8-7981 | NA | 4 | Yes |  |  |  |  |
| 24 | Braenderup | FSL R8-7984 | NA | 4 | Yes |  |  |  |  |
| 25 | Paratyphi B var. Java | FSL S5-0447 | human | 4 | Yes | Yes |  |  |  |
| 26 | Mbandaka | FSL S5-0451 | human | 4 | Yes |  |  |  |  |
| 27 | Virchow | FSL S5-0961 | bovine | 4 | Yes | Yes |  |  |  |
| 28 | Typhi | FSL R6-0540 | human, clinical | 5 | Yes |  |  |  |  |
| 29 | Derby | FSL R8-2630 | NA | 5 | Yes |  |  |  |  |
| 30 | Heidelberg | FSL S5-0448 | human | 5 | Yes |  |  |  |  |
| 31 | Thompson | FSL S5-0523 | bovine | 5 | Yes |  |  |  |  |
| 32 | 4,[5],12:i:- | FSL S5-0580 | bovine | 5 | Yes |  | Yes |  |  |
| 33 | Montevideo | FSL S5-0630 | bovine | 5 | Yes |  |  |  |  |
| 34 | Saintpaul | FSL S5-0649 | human | 5 | Yes |  |  |  |  |
| 35 | Newport | FSL R8-7979 | NA | 6 | Yes |  |  |  |  |
| 36 | Agona | FSL S5-0517 | human | 6 | Yes |  |  |  |  |
| 37 | Infantis | FSL S5-0734 | bovine | 7 | Yes |  |  |  |  |
| 38 | Enteritidis | FSL S5-0415 | human | 8 | Yes |  |  |  |  |
| 39 | Typhimurium | FSL S5-0536 | human | 8 | Yes |  | Yes |  |  |
| 40 | Putten | FSL A4-0590 | human | 0 |  |  |  |  |  |
| 41 | Poona | FSL R8-0115 | human, clinical | 0 |  |  |  | Yes |  |
| 42 | Pomona | FSL R8-0451 | human, clinical | 0 |  |  |  |  |  |
| 43 | Barranquilla | FSL R8-1295 | human, clinical | 0 |  |  |  |  |  |
| 44 | Minnesota | FSL R8-2410 | animal, non-clinical | 0 |  |  |  |  | Yes |
| 45 | Ealing | FSL R8-2454 | human, clinical | 0 |  |  |  |  |  |
| 46 | Alachua | FSL R8-2924 | human, clinical | 0 |  |  |  |  |  |
| 47 | Uganda | FSL R8-3404 | animal, clinical | 0 |  |  |  |  |  |
| 48 | Cubana | FSL R8-3581 | human, clinical | 0 |  |  |  |  | Yes |
| 49 | Typhimurium o5- | FSL R8-3714 | human, clinical | 0 |  |  | Yes |  |  |
| 50 | Orion var 15+, 34+ | FSL R8-3858 | animal, clinical | 0 |  | Yes |  |  |  |
| 51 | Ohio | FSL R8-4333 | NA | 0 |  |  |  |  |  |
| 52 | Ibadan | FSL R8-4726 | NA | 0 |  |  |  |  |  |
| 53 | Stockholm | FSL R8-4727 | NA | 0 |  |  |  |  |  |
| 54 | Apapa | FSL R8-5222 | NA | 0 |  |  |  |  |  |
| 55 | Hartford | FSL R8-5223 | NA | 0 |  |  |  |  |  |
| 56 | Gaminara | FSL R8-5569 |  | 0 |  |  |  |  |  |
| 57 | Norwich | FSL R8-6279 | NA | 0 |  |  |  |  |  |
| 58 | Meleagridis | FSL R8-6670 | NA | 0 |  |  |  |  |  |
| 59 | Roodepoort | FSL R8-7983 | NA | 0 |  |  |  |  |  |
| 60 | Kiambu | FSL R8-9562 | NA | 0 |  |  |  |  |  |
| 61 | S. enterica subspecies IIIa -:z4,z23:- | FSL R9-0515 | NA | 0 |  |  |  |  |  |
| 62 | S. enterica subspecies IIIb 6,7:l,v:z53 | FSL R9-0516 | NA | 0 |  |  |  |  |  |
| 63 | S. enterica subspecies IV 45:g,z51:- | FSL R9-0517 | NA | 0 |  |  |  |  |  |
| 64 | S. bongori subspecies V 66:z41:- | FSL R9-0518 | NA | 0 |  |  |  | Yes |  |
| 65 | Liverpool | FSL R9-1184 | human, clinical | 0 |  |  |  |  | Yes |
| 66 | S. enterica subspecies VI [1],6,14,[25]:a:e,n,x | FSL R9-8566 | NA | 0 |  |  |  |  |  |
| 67 | Give | FSL S5-0487 | human | 0 |  | Yes |  |  |  |
| 68 | Havana | FSL S5-0549 | animal | 0 |  |  |  |  | Yes |
| 69 | Johannesburg | FSL S5-0703 | human | 0 |  |  |  |  | Yes |

**Supplementary Table 2. Data distribution of each multiplexed isolates in each flow cell.**

| **Group/Flow cell No.** | **Barcode ID** | **Number of reads** | **Total clean data yield in 24h (Gbp)** | **Average read length** | **Proportion of the total flowcell data yield(%)** | **Estimated depth of genome coverage  (Assuming genome size of Salmonella is 4.8 Mbp)** |
| --- | --- | --- | --- | --- | --- | --- |
| 1 | No. 01 | 183,164 | 1,434,971,570 | 7,834 | 21.40 | 298.95 |
| 1 | No. 02 | 91,015 | 983,547,063 | 10,806 | 14.67 | 204.91 |
| 1 | No. 03 | 70,642 | 742,291,479 | 10,508 | 11.07 | 154.64 |
| 1 | No. 04 | 189,669 | 1,548,680,526 | 8,165 | 23.10 | 322.64 |
| 1 | No. 05 | 194,876 | 1,485,615,496 | 7,623 | 22.16 | 309.50 |
| 1 | None-assigned | 56,711 | 507,738,739 | 8,953 | 7.57 | NA |
| 1 | mis-assigned | 232 | 1,928,597 | 9,739 | 0.03 | NA |
| 2 | No. 01 | 151,678 | 1,133,429,898 | 7,473 | 18.38 | 236.13 |
| 2 | No. 02 | 137,662 | 1,300,456,140 | 9,447 | 21.09 | 270.93 |
| 2 | No. 03 | 86,200 | 722,615,330 | 8,383 | 11.72 | 150.54 |
| 2 | No. 04 | 155,846 | 1,572,088,075 | 10,087 | 25.49 | 327.52 |
| 2 | No. 05 | 120,209 | 1,013,239,763 | 8,429 | 16.43 | 211.09 |
| 2 | None-assigned | 45,680 | 423,581,577 | 9,273 | 6.87 | NA |
| 2 | mis-assigned | 185 | 1,515,372 | 8,511 | 0.02 | NA |
| 3 | No. 01 | 175,823 | 1,252,450,417 | 7,123 | 17.42 | 260.93 |
| 3 | No. 02 | 181,719 | 1,695,334,861 | 9,329 | 23.58 | 353.19 |
| 3 | No. 03 | 106,289 | 903,878,785 | 8,504 | 12.57 | 188.31 |
| 3 | No. 04 | 122,847 | 970,463,996 | 7,900 | 13.50 | 202.18 |
| 3 | No. 05 | 230,167 | 1,890,692,663 | 8,214 | 26.30 | 393.89 |
| 3 | None-assigned | 55,449 | 474,569,823 | 8,559 | 6.60 | NA |
| 3 | Cross-assigned | 276 | 2,454,531 | 9,764 | 0.03 | NA |
| 4 | No. 01 | 95,330 | 947,333,659 | 9,937 | 14.18 | 197.36 |
| 4 | No. 02 | 155,125 | 1,587,262,893 | 10,232 | 23.76 | 330.68 |
| 4 | No. 03 | 90,306 | 879,612,253 | 9,740 | 13.16 | 183.25 |
| 4 | No. 04 | 185,492 | 1,722,145,933 | 9,284 | 25.77 | 358.78 |
| 4 | No. 05 | 124,047 | 1,117,849,160 | 9,012 | 16.73 | 232.89 |
| 4 | None-assigned | 43,425 | 425,729,321 | 9,804 | 6.37 | NA |
| 4 | mis-assigned | 188 | 1,698,987 | 10,134 | 0.03 | NA |
| 5 | No. 01 | 188,742 | 1,557,094,737 | 8,250 | 21.65 | 324.39 |
| 5 | No. 02 | 78,197 | 971,172,340 | 12,420 | 13.50 | 202.33 |
| 5 | No. 03 | 66,612 | 556,021,154 | 8,347 | 7.73 | 115.84 |
| 5 | No. 04 | 259,390 | 2,293,091,629 | 8,840 | 31.88 | 477.73 |
| 5 | No. 05 | 161,931 | 1,234,991,589 | 7,627 | 17.17 | 257.29 |
| 5 | None-assigned | 64,786 | 577,928,041 | 8,921 | 8.04 | NA |
| 5 | mis-assigned | 176 | 1,647,674 | 11,140 | 0.02 | NA |
| 6 | No. 01 | 71,911 | 647,782,864 | 9,008 | 12.20 | 134.95 |
| 6 | No. 02 | 105,988 | 850,194,638 | 8,022 | 16.02 | 177.12 |
| 6 | No. 03 | 82,984 | 941,870,014 | 11,350 | 17.74 | 196.22 |
| 6 | No. 04 | 139,317 | 1,464,983,433 | 10,516 | 27.60 | 305.20 |
| 6 | No. 05 | 131,595 | 1,003,724,980 | 7,627 | 18.91 | 209.11 |
| 6 | None-assigned | 41,761 | 398,324,277 | 9,538 | 7.50 | NA |
| 6 | mis-assigned | 153 | 1,345,238 | 10,073 | 0.03 | NA |
| 7 | No. 01 | 106,132 | 909,862,916 | 8,573 | 15.43 | 189.55 |
| 7 | No. 02 | 185,930 | 1,829,113,262 | 9,838 | 31.02 | 381.07 |
| 7 | No. 03 | 81,331 | 795,770,967 | 9,784 | 13.50 | 165.79 |
| 7 | No. 04 | 122,080 | 1,133,817,298 | 9,288 | 19.23 | 236.21 |
| 7 | No. 05 | 100,317 | 813,226,847 | 8,107 | 13.79 | 169.42 |
| 7 | None-assigned | 44,609 | 413,090,784 | 9,260 | 7.01 | NA |
| 7 | mis-assigned | 198 | 1,647,006 | 10,773 | 0.03 | NA |
| 8 | No. 01 | 225,635 | 1,744,226,866 | 7,730 | 22.84 | 363.38 |
| 8 | No. 02 | 216,538 | 2,018,504,264 | 9,322 | 26.43 | 420.52 |
| 8 | No. 03 | 65,385 | 495,280,180 | 7,575 | 6.49 | 103.18 |
| 8 | No. 04 | 133,377 | 1,425,096,763 | 10,685 | 18.66 | 296.90 |
| 8 | No. 05 | 170,959 | 1,369,284,871 | 8,009 | 17.93 | 285.27 |
| 8 | None-assigned | 60,881 | 581,885,656 | 9,558 | 7.62 | NA |
| 8 | mis-assigned | 313 | 2,591,462 | 10,474 | 0.03 | NA |
| 9 | No. 01 | 115,980 | 921,775,551 | 7,948 | 15.94 | 192.04 |
| 9 | No. 02 | 70,522 | 685,827,738 | 9,725 | 11.86 | 142.88 |
| 9 | No. 03 | 180,773 | 1,591,668,424 | 8,805 | 27.53 | 331.60 |
| 9 | No. 04 | 123,461 | 984,378,685 | 7,973 | 17.03 | 205.08 |
| 9 | No. 05 | 144,562 | 1,168,209,196 | 8,081 | 20.21 | 243.38 |
| 9 | None-assigned | 50,183 | 427,715,226 | 8,523 | 7.40 | NA |
| 9 | mis-assigned | 228 | 1,790,101 | 9,067 | 0.03 | NA |
| 10 | No. 01 | 113,973 | 1,185,270,267 | 10,400 | 16.47 | 246.93 |
| 10 | No. 02 | 68,907 | 1,023,398,831 | 14,852 | 14.22 | 213.21 |
| 10 | No. 03 | 79,235 | 967,808,135 | 12,214 | 13.45 | 201.63 |
| 10 | No. 04 | 152,447 | 1,265,797,571 | 8,303 | 17.59 | 263.71 |
| 10 | No. 05 | 229,128 | 2,289,251,832 | 9,991 | 31.81 | 476.93 |
| 10 | None-assigned | 43,089 | 462,419,878 | 10,732 | 6.43 | NA |
| 10 | mis-assigned | 222 | 2,500,276 | 12,264 | 0.03 | NA |
| 11 | No. 01 | 220,881 | 1,752,133,958 | 7,933 | 21.02 | 365.03 |
| 11 | No. 02 | 215,739 | 1,781,210,136 | 8,256 | 21.36 | 371.09 |
| 11 | No. 03 | 105,022 | 952,722,668 | 9,072 | 11.43 | 198.48 |
| 11 | No. 04 | 155,557 | 1,383,860,519 | 8,896 | 16.60 | 288.30 |
| 11 | No. 05 | 213,422 | 1,864,541,316 | 8,736 | 22.36 | 388.45 |
| 11 | None-assigned | 71,537 | 600,402,433 | 8,393 | 7.20 | NA |
| 11 | mis-assigned | 282 | 2,466,751 | 11,236 | 0.03 | NA |
| 12 | No. 01 | 89,106 | 1,001,641,398 | 11,241 | 17.63 | 208.68 |
| 12 | No. 02 | 62,478 | 642,875,874 | 10,290 | 11.31 | 133.93 |
| 12 | No. 03 | 68,399 | 789,099,389 | 11,537 | 13.89 | 164.40 |
| 12 | No. 04 | 89,573 | 1,030,918,646 | 11,509 | 18.14 | 214.77 |
| 12 | No. 05 | 163,602 | 1,766,084,975 | 10,795 | 31.08 | 367.93 |
| 12 | None-assigned | 39,394 | 449,095,109 | 11,400 | 7.90 | NA |
| 12 | mis-assigned | 174 | 1,951,336 | 12,728 | 0.03 | NA |
| 13 | No. 01 | 183,495 | 1,586,602,481 | 8,647 | 20.37 | 330.54 |
| 13 | No. 02 | 200,849 | 2,023,250,758 | 10,074 | 25.97 | 421.51 |
| 13 | No. 03 | 80,903 | 1,073,313,305 | 13,267 | 13.78 | 223.61 |
| 13 | No. 04 | 90,843 | 1,087,570,007 | 11,972 | 13.96 | 226.58 |
| 13 | No. 05 | 147,957 | 1,467,042,475 | 9,915 | 18.83 | 305.63 |
| 13 | None-assigned | 51,801 | 550,342,585 | 10,624 | 7.06 | NA |
| 13 | mis-assigned | 222 | 2,544,626 | 13,544 | 0.03 | NA |
| 14 | No. 01 | 79,793 | 710,431,587 | 8,903 | 15.74 | 148.01 |
| 14 | No. 02 | 40,557 | 499,014,701 | 12,304 | 11.06 | 103.96 |
| 14 | No. 03 | 48,940 | 533,310,105 | 10,897 | 11.82 | 111.11 |
| 14 | No. 04 | 208,123 | 1,776,516,423 | 8,536 | 39.36 | 370.11 |
| 14 | No. 05 | 65,318 | 669,940,917 | 10,257 | 14.84 | 139.57 |
| 14 | None-assigned | 33,544 | 323,450,829 | 9,643 | 7.17 | NA |
| 14 | mis-assigned | 108 | 1,169,916 | 11,515 | 0.03 | NA |
| - | Cross-assigned  (Average No = 14) | 211 | 1,946,562 | 10,783 | 0.03 | - |
| - | None-assigned  (Average No = 14)) | 50,204 | 472,591,020 | 9,513 | 7.20 | - |
